# Retrospective analysis of *Clostridioides difficile* and other intestinal infections in patients with Crohn’s disease and ulcerative colitis in the tertiary hospital in Poland. POLIBD survey results

**DOI:** 10.1186/s13099-021-00471-z

**Published:** 2021-12-13

**Authors:** Jolanta Gruszecka, Rafał Filip

**Affiliations:** 1 Institute of Health Sciences, Medical College of Rzeszow University, Rzeszow, Poland; 2 Department of Clinical Microbiology, Clinical Hospital No. 2, im. Św. Jadwigi Królowej, Rzeszow, Poland; 3 Department of Gastroenterology with IBD Unit of Clinical Hospital No. 2 Im. Św. Jadwigi Królowej, Rzeszow, Poland; 4 Faculty of Medicine, University of Rzeszow, Rzeszow, Poland

**Keywords:** Intestinal infections, *Clostridioides difficile*, Risk factor, Inflammatory bowel disease

## Abstract

**Background:**

There are several studies which evaluated the number of infections caused by enteric pathogens, including *Clostridioides difficile* in patients with inflammatory bowel disease (IBD). Our aim was to assess the prevalence of intestinal infections among patients suffering from IBD, when admitted to the hospital due to exacerbation of the disease.

**Results:**

The performed, retrospective analysis covered test results for *C. difficile* toxins A and B along with rectal swab cultures sampled from patients, treated in a tertiary IBD center in Poland, between 2017 and 2019. Main objective was to estimate the presence of any infection, which could imitate or co-exist along with the exacerbation of the IBD. All in all 1471 patients had microbiological tests performed, including 1112 tested for *C. difficile* toxins A and B; and 359 patients who had rectal swab culture. Positive test results for *C. difficile* toxins A and B were reported in 358 cases, positive results from rectal swab culture were confirmed altogether in case of 25 samples. As far as patients with IBD are concerned, positive results for *C. difficile* toxins A and B were detected in 82 cases, positive results in rectal swab culture from patients with IBD were reported in 20 cases.

**Conclusion:**

Intestinal infections were reported in 14.9% of patients (102/685) with IBD symptoms. Positive test results for *C. difficile* toxins A and B and rectal swab cultures among patients without IBD symptoms were reported in 35.7% of cases (281/786). Intestinal superinfections may complicate the clinical picture of IBD patients, increasing the diagnostic and therapeutic burden. Appropriate early procedures are thus needed in these patients.

## Introduction

Inflammatory bowel diseases are diseases in the course of which there is chronic inflammation of the gastrointestinal tract. Their conditions are not fully understood, but it is known that immunological, genetic and environmental factors play a role in the pathogenesis of the disease state. Allergic factors, bacterial and viral infections are also taken into account. The group of inflammatory bowel diseases include Crohn’s disease (CD) and ulcerative colitis (UC) [1].

*Clostridioides difficile* is a Gram-positive, spore-forming, strictly anaerobic bacillus which was first isolated from the stool of a healthy infant by Hall and O’Toole in 1935 [2]. They were first identified in the 1970s. [3].

*Clostridioides difficile* can exist in vegetative or spore form. In its spore form, the bacterium can survive harsh environments and common sterilization techniques. Spores of *C. difficile* are resistant to high temperatures, ultraviolet light, harsh chemicals, and antibiotics. Because the spores are resistant to antibiotics, they can remain in the gastrointestinal tract and potentially contribute to recurrent disease following treatment and eradication of vegetative *C. difficile*. Pathogenic *C. difficile* organisms release 2 potent toxins A i B, that ultimately mediate diarrhoea and colitis [2, 4]. This bacterium can be a normal part of the intestinal microflora detected in healthy individuals, but without causing disease by its presence (asymptomatic carrierstate affects approximately 3% of adults and two-thirds of children) [5].

The results of many studies have demonstrated that diarrheal relapses of inflammatory bowel disease (IBD) may be associated or confound with various enteric infections. Moreover, existing data suggesting that gastrointestinal infections may be associated with later development of inflammatory bowel diseases [6–8].

Bacterial infections were more common with parasitic and viral infections less common in patients with ulcerative colitis (UC) when compared to non-IBD patients [8]. On the other hand, the significance of previous GI infections in the development of both Crohn’s disease (CD) and UC was shown in a nationwide case–control study performed in Sweden. Moreover, the authors concluded that enteric infections might induce microbial dysbiosis that contributes to the development of IBD in susceptible individuals [9]. It is also worth mentioning that GI infections, *Clostridioides difficile* infections (CDIs) in particular, were associated with longer hospitalizations, higher hospital costs, and greater overall mortality [7–11].

Since there is no data on the prevalence of *Clostridioides difficile* infections in IBD patients during flare-ups in Eastern Europe, in this study we aimed to investigate the incidence of concurrent infection in Polish patients reporting a relapse over a recent 3-year period. Stool microbiology results routinely obtained during hospital admission relating to relapses of IBD throughout the 2017–2019 period were obtained retrospectively.

## Material and methods

A retrospective analysis was made test results of adult patients admitted and then treated in a tertiary IBD center in Rzeszów (southern Poland) between the 1st of January, 2017, and the 31st of December, 2019. Data of all hospitalized patients used for the purpose of the analysis were collected from the electronic medical documentation kept by the hospital. The identification of patients with IBD was based on the international classification of Crohn’s disease or ulcerative colitis.

The study was approved by the Bioethics Committee of the Regional Medical Chamber (Resolution No. 88/B/2020 of September 24, 2020).

As only a retrospective study was performed, according to Polish law, patient consent was not required.

Stool samples obtained from patients treated in a tertiary IBD center in Rzeszów were tested for *Clostridioides difficile* toxins A and B. Rectal swab cultures were also performed. Patients diagnosed with Crohn's disease or ulcerative colitis admitted due to suspicion of exacerbation or with symptoms such as fever, stomachache, hematochezia, or diarrhea (determined by an increased average daily number of defecations ¥3 times a day) were enrolled in the trial [12]. Collected samples were then delivered to the Clinical Microbiological Laboratory.

Tests for *C. difficile* toxins A and B were performed with a chemiluminescent immunoassay (CLIA) intended for the qualitative determination of *Clostridioides difficile* toxins A and B in human feces on the LIAISON Analyzer (DiaSorin S.p.A. Italy). The test used detects *C. difficile* A and/or B toxins in the stool samples. Rectal swabs were collected before the initiation of the treatment and were processed according to current binding methodologies. Identification of the cultured microorganisms was carried out by mass spectroscopy (MALDI-TOF MS) using an automatic mass spectrometer VITEK MS (bioMérieux, France) [13]. The drug resistance profile of cultured and identified microorganisms was determined by the disc diffusion method, or means of a VITEK2 (bioMérieux, France) automatic system for identification and determination of susceptibility according to EUCAST (European Committee on Antimicrobial Susceptibility Testing) [14].

Patients who had a CDIs infection in the past or who were hospitalized for the period of 3–6 months were tested for *C. difficile* toxins A and B. Patients without a history of CDI and without previous hospitalization had rectal swab cultures performed. There was no other enteric organism present on the rectal swabs other than that mentioned, and this was the reason for further characterization of the isolate.

## Results

A total of 1471 patients treated in a tertiary IBD center in Poland were examined between the 1st of January, 2017, and the 31st of December, 2019. They had their stool tested for *C. difficile* toxins A and B, and they had rectal swab culture performed.

**Fig. 1:**
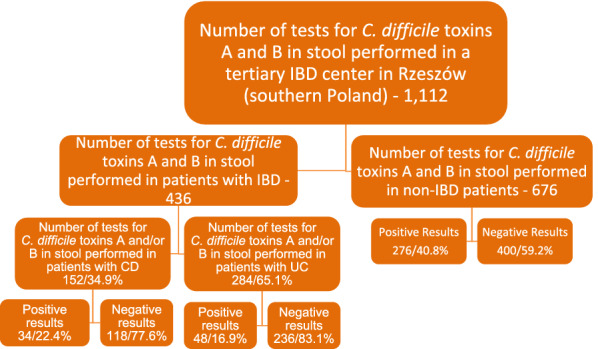
Percentages of positive and negative results for *C. difficile* toxins A and B in the population sampled

Altogether, 1112 patients were tested for the presence of *C. difficile* toxins A and B; 359 patients had a rectal swab culture performed. Positive results for *C. difficile* toxins A and B were reported in 32.2% of cases (358/1112), including 82 patients with IBD (Fig. 1).

**Fig. 2:**
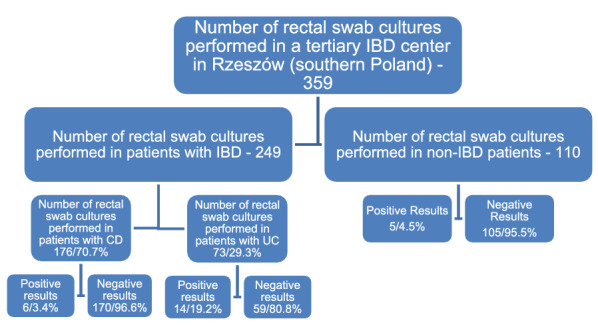
Percentages of positive and negative results of cultures for microorganism recovery in the population sampled

Positive results of rectal swab culture were confirmed in 6.96% of samples (25/359), including 20 patients suffering from IBD (Fig. 2).

A summary of the cultures of rectal swabs of IBD and non-IBD patients is presented in Table 1.

**Table 1:** Rectal swab culture results in patients with IBD and non-IBD patients in a tertiary center in Rzeszow (Southern Poland) along with cultured microorganisms (January 2017—December 2019)

Number of rectal swabs (n)	Positive results (n/%)	Cultured microorganisms	Number	% in relation to all samples taken	% in relation to positive results
Patients with IBD					
249	20/9.8	*Candida albicans*	14	5.6	70.0
		*Candida* sp. non *albicans*	2	0.8	10.0
		*Candida glabrata*	2	0.8	10.0
		*Candida lusitaniae*	1	0.4	5.0
		*Candida kefyr*	1	0.4	5.0
Non-IBD patients					
110	5/4.54	*Candida albicans*	1	0.91	20.0
		*Candida glabrata*	2	1.82	40.0
		*Salmonella enterica*	2	1.82	40.0

The drug resistance profile of cultured and identified microorganisms was mentioned in Table 2.

**Table 2:** Rectal swab culture results in patients with IBD and non-IBD patients in a tertiary center in Rzeszow (Southern Poland) along with cultured microorganisms (January 2017—December 2019)

Number of rectal swabs, n	Positive results, n/%	Cultured microorganisms	Number	Susceptibility of the cultured microorganisms^a^	% in relation to all collected samples	% in relation to positive results
Patients with IBD						
249	20/9.8	*Candida albicans*	14	AmB(s), CAS(s), FLU(s), AFY(s), MYC(s), VO(s)	5.6	70.0
				AmB(s), CAS(s), FLU(s), AFY(s), MYC(s), VO(s)		
				AmB(s), CAS(s), FLU(s), AFY(s), MYC(s), VO(s)		
				AmB(s), CAS(s), FLU(s), MYC(s), VO(s)		
				AmB(s), CAS(s), FLU(s), AFY(s), MYC(s), VO(s)		
				AmB(s), CAS(s), FLU(s), AFY(s), MYC(s), VO(s)		
				AmB(s), CAS(s), FLU(s), AFY(s), MYC(s), VO(r)		
				AmB(s), CAS(s), FLU(s), AFY(s), MYC(s), VO(s)		
				AmB(s), CAS(s), FLU(s), AFY(s), MYC(s), VO(s)		
				AmB(s), CAS(s), FLU(s), AFY(s), MYC(s), VO(s)		
				AmB(s), CAS(s), FLU(s), AFY(s), MYC(s), VO(s)		
				AmB(s), CAS(s), FLU(s), AFY(s), MYC(s), VO(s)		
				AmB(s), CAS(s), FLU(s), AFY(s), MYC(s), VO(r)		
				AmB(s), CAS(s), FLU(s), AFY(s), MYC(s), VO(s)		
		*Candida* sp. non *albicans*	2	AmB(s), CAS(s), FLU(s), MYC(s), VO(s)	0.8	10.0
				AmB(s), CAS(s), FLU(s), AFY(s), MYC(s), VO(s)		
		*Candida glabrata*	2	AmB(s), CAS(s), FLU(s), MYC(s)	0.8	10.0
				AmB(s), CAS(s), FLU(s), MYC(s)		
		*Candida lusitaniae*	1	AmB(s), CAS(s), FLU(s), AFY(s), MYC(s), VO(s)	0.4	5.0
		*Candida kefyr*	1	AmB(s), CAS(s), FLU(s), AFY(s), MYC(s), VO(s)	0.4	5.0
Non-IBD patients						
110	5/4.54	*Candida albicans*	1	AmB(s), CAS(s), FLU(s), AFY(s), MYC(s), VO(s)	0.91	20.0
		*Candida glabrata*	2	AmB(s), CAS(s), FLU(s), MYC(s)	1.82	40.0
				AmB(s), CAS(s), FLU(r), MYC(s)		
		*Salmonella enterica*	2	AK(s), SAM(s), FEP(s), CTX(s), CAZ(s), CXM(s), CIP(s), CT(s), ETP(s), GM(s), IPM(s), MEM(s), TZP(s), TGC(s), SXT(s)	1.82	40.0
				AK(s), SAM(s), FEP(s), CTX(s), CAZ(s), CXM(s), CIP(r), CT(s), ETP(s), GM(s), IPM(s), MEM(s), TZP(s), TGC(s), SXT(s)		

^a^s—sensitive, r—resistant

*AmB* amphotericin B, *CAS* caspofungin, *FLU* fluconazole, *AFY* flucytosine, *MYC* micafungin, *VO* voriconazole, *AK* amikacin, *SAM* ampicillin/sulbactam, *FEP* cefepime, *CTX* cefotaxime, *CAZ* ceftazidime, *CXM* cefuroxime, *CIP* ciprofloxacin, *CT* colistin, *ETP* ertapenem, *GM* gentamicin, *IPM* imipenem, *MEM* meropenem, *TZP* piperacillin/tazobactam, *TGC* tigecycline, *SXT* trimethoprim /sulfamethoxazole

During the analyzed period 436 tests for the presence of *C. difficile* toxins A and B were performed in patients with IBD. Positive results were reported in 44 women (53.7%) and 38 men (46.3%).

The evaluation concerning obtained results enables us to observe that positive rectal swab culture in patients with IBD was much more frequently reported in samples collected from women (13 samples—65.0%) than in the ones collected from men (7 samples—35.0%).

The analysis focused on test results for *C. difficile* toxins A and B in the stool of patients suffering from inflammatory bowel disease, treated in a tertiary IBD center in Poland, revealed a seasonal variability. In the summer months, there were 60 positive results, whereas in the winter months the number of positive results was far lower, 22 (Table 3).

**Table 3:** Seasonal variability in the occurrence of positive test results in patients with IBD

Summer months	Winter months
Positive results of tests for *C. difficile* toxins A and B in stool	
60	22
Positive results of rectal swab cultures	
14	6

The analysis of obtained microbiological results from the examined rectal swab cultures in patients with IBD also showed a seasonal variability. Positive rectal swab cultures sampled from patients with IBD were more frequently observed in summer months, 14, than in the winter months, when only 6 positive test results were reported (Table 3).

Clinical information on patients with IBD is presented in Table 4.

**Table 4:** Clinical charakteristics of study population

Patients’ characteristics	CD			UC		
	Negative results for *C. difficile* toxins A and/or B tests	Positive results for *C. difficile* toxins A and/or B tests	Positive results of rectal swab cultures	Negative results for* C. difficile* toxins A and/or B tests	Positive results for *C. difficile* toxins A and/or B tests	Positive results of rectal swab cultures
Vomiting, n (%)	58 (49)	22 (64.7)	3 (50)	–	4 (8.3)	1 (7.1)
Diarrhea, n (%)	54 (45.8)	30 (88.2)	6 (100)	224 (95)	48 (100)	14 (100)
Fever, n (%)	33 (28)	11 (32.3)	4 (66.7)	47 (19.9)	15 (31.2)	4 (28.6)
Antibiotic therapy implemented, n (%)	12 (10.7)	34 (100)	6 (100)	35 (14.9)	48 (100)	13 (92.8)
Stomach pain, n (%)	106 (89.8)	34 (100)	1 (16.7)	165 (69.9)	47 (97.9)	1 (7.1)
The onset of the disease before admission to the hospital	2–8 weeks	1–6 weeks		2–4 weeks	1–12 weeks	
Taking samples for research	All samples were taken during hospitalization					

## Discussion

Patients suffering from inflammatory bowel disease are more prone to increased risk of intestinal infection [15, 16]. Risk factors associated with CDIs traditionally include age, use of antibiotics, severe co-morbidities, next to contact with a hospital, or other primary care facilities [17].

Multiple crucial factors that increase the risk of CDIs include the use of broad-spectrum antibiotics that damage the intestinal microflora and create conditions for the multiplication of pathogenic microorganisms [18]. Risk factors for CDIs associated with hospitalization also include: use of immunosuppressive medications, cytostatics, co-morbidities and any conditions related to diseases that contribute to the occurrence of endogenous infections, surgical procedures within the alimentary tract, long hospitalization, improper medical procedures related with the patient’s stay in the hospital, as well as sanitary conditions [18, 19].

*Clostridioides difficile* infections have become a particular problem for patients with IBD. Patients with IBD have an increased risk of poor outcomes when suffering from CDIs, associated with a higher frequency of flare-ups, greater morbidity and mortality, poorer response to treatment, need for more active treatment for IBD, and longer duration of hospital stay [20, 21].

Several prospective observational studies and case reports have highlighted the predisposition of patients with IBD to develop severe infections due to opportunistic and common microbial pathogens [22, 23]. Opportunistic infections are associated with significant mortality and morbidity in individuals with a compromised immune system [24].

The risk factors for opportunistic infections are malnutrition, older age, congenital immunodeficiency, HIV infection, chronic diseases (such as emphysema), diabetes mellitus, and use of immunosuppressive medications such as corticosteroids, immunomodulators (methotrexate, thiopurines), and anti-TNF-a therapy [25, 26]. Quick start of biological therapy may be safer if new drugs are used: Tofacitinip, Vedolizumap. Acting selectively, they affect the functioning of the immune system, humoral and cellular response, unlike drugs from the anti-TNF-α group, which significantly increase the risk of opportunistic infections.

Opportunistic infections in patients with IBD include viral infections (herpes viruses, human papillomavirus, influenza virus, and JC virus), bacterial infections (tuberculosis, nocardiosis, *Clostridioides difficile* infection, pneumococcal infection, legionellosis, and listeriosis), fungal infections (histoplasmosis, cryptococcosis, *Pneumocystis jirovecii* infection, aspergillosis, and candidiasis), and parasite infections (*Strongyloides stercoralis*) [27].

Cytomegalovirus (CMV) infection is one of the most common viral infections and can occur both as a co-infection and in an isolated manner. In the endoscopic image, it resembles an exacerbation of ulcerative colitis [12, 28].

The European Crohn’s and Colitis Organisation (ECCO) states that all patients with IBD on corticosteroids, immunomodulators, and biological agents should be considered immunocompromised and at risk for opportunistic infections [10].

Epidemiological data, although sparse, clearly show that the incidence of GI infections in IBD patients is increasing with time. A US study showed that the main driver of this rising incidence was *C. difficile* infections, which, in that particular study, increased from 7.8 to 32.1 per 1000 Crohn’s disease hospitalizations and from 23.0 to 84.7 per 1000 ulcerative colitis hospitalizations [7]. In the same study the incidence of other intestinal infections increased from 10.2 to 15.3 per 1000 CD hospitalizations and 16.5–25.3 per 1000 UC hospitalizations [7]. In another study, which involved approximately 9000 patients, of which 577 were IBD patients, non-*Clostridium difficile* enteric infections were identified in 17% of symptomatic patients with IBD. Compared with non-IBD patients, CD patients had a higher prevalence of norovirus and *Campylobacter* and a lower prevalence of parasites [8].

A study conducted in North America between the years 2004–2010 focused on outcomes resulting from an anti-TNF therapy in 6273 patients suffering from Crohn’s disease. The average observation time in all patients was 5.2 years [23]. Within the observed group of patients the researchers observed 6 severe fungal infections (caused by the presence of *Candida glabrata* and *Candida tropicalis)*, as well as 6 *C. difficile* infections [23].

Another 5-year-long French study, conducted in the years 2009–2014 on a cohort of 190,694 patients suffering from IBD, revealed the presence of 8561 severe infections and 674 opportunistic infections; whereas 160 cases of the above-mentioned infections were bacterial infections and 76 were fungal infections. Candidiasis was reported in 36 cases [29].

The analysis performed in a tertiary Italian hospital (Perugia—central Italy) showed the occurrence of intestinal superinfections in patients with intestinal inflammations reported between June 2007 and June 2010. All in all, samples for microbiological and parasitological tests were collected from 98 patients without symptoms of infections and from 15 patients with infection. The analyzed material revealed the presence of *C. difficile* (7 cases), *Campylobacter jejuni* (3 cases), and Cytomegalovirus (CMV) (7 cases). There were no other intestinal superinfections observed [28].

In our study that was conducted in a tertiary hospital in Rzeszow (southern Poland) between January 2017 and December 2019 an overall number of 1471 tests were performed, including 1112 tests for the presence of *C. difficile* toxins A and B in stool, along with 359 rectal swab cultures. The presence of toxins A and B in stool was reported in 18.8% of samples (82/436) collected from patients with IBD and in 40.8% of samples (276/676) collected from non-IBD patients. (Fig. 1). Rectal swab culture was performed for 249 patients with IBD and 110 non-IBD patients (Fig. 2). The growth of fungi from the *Candida* family was noted in 8.0% of samples (20/249) obtained from patients with IBD, whereas in the material sampled from non-IBD patients *Candida albicans* (3 cases) and *Salmonella enterica* (2 cases) were grown (Table 1).

Our study found an increase in the number of positive microbiological test results in the summer months. This may be favored by higher ambient temperature, lifestyle changes: more frequent travel and related changes in eating and hygiene habits, sometimes access to poorer quality water, gastrointestinal infections, acquisition of endemic infectious diseases, as well as difficult access to medications [30].

Infections caused by *C. difficile* strains stand as one of the main factors responsible for the prolonged hospitalization of patients. According to the American and European data, each year the number of CDIs ranges from 10 to 90 cases per 10,000 hospitalizations [31]. In the USA CDIs cause nearly half a million infections every year, and it has been estimated that the cost of treatment is close to $4.8 billion per year [32].

In Poland, the number of infections caused by *C. difficile* has more than doubled between 2013 and 2018. In 2013 the morbidity rate in Poland equaled 12.3 per 100,000 inhabitants (4728 cases), whereas in the year 2018 it reached 30.2 per 100,000 (11,592 cases) [33].

In the UK the frequency of *C. difficile* infections in 2018 equaled 24 cases per 100,000 residents, a total of 13,286 diagnosed cases of CDIs [32].

In France, it has been estimated that the average cost of treating a patient with CDIs is EUR 9575, which stands that an additional EUR 163.1 millions needed to be transferred by the state to the health care system [34]. In Germany, the additional costs allocated to treating a patient with recurring *C. difficile* infection increased to EUR 7654 [35].

European analysis revealed that the overall costs related to treating a patient with CDIs amount to EUR 33,840. These costs are going to increase annually due to the progression of the aging demographic [19].

According to Ahmad S. and Khan Z., invasive *Candida* infections are mainly caused by four species, including *C. albicans*, *C. glabrata*, *C. parapsilosis*, and *C. tropicalis* [36]. Immunosuppression in IBD is associated with oral, esophageal, or systemic candidiasis, although there is currently no specific recommendation for screening and prophylaxis [10, 23].

In our study, during the observation period, 23 cases had the presence of *Candida* species in rectal swab cultures (20—IBD patients, 3—non-IBD patients). These were: *C. albicans*, *C. glabrata*, *C. lusitaniae, C. kefyr, C.* sp. non *albicans* (Table 1)*.*

In conclusion, intestinal superinfections in IBD patients, although relatively infrequent, increase co-morbidity, aggravate the diagnostic and therapeutic burden of these subjects, in addition, raise sanitary costs, especially in light of new therapeutic approaches. Thus, early recognition of complicating infections with a targeted therapeutic approach is needed and probably could ensure a better prognosis for IBD patients [37].

Our research is a retrospective study, and more reliable conclusions should be clarified by further prospective studies.

## Data Availability

The datasets during and/or analysed during the current study available from the corresponding author on reasonable request.
